# The *Shigella flexneri* virulence factor apyrase is released inside eukaryotic cells to hijack host cell fate

**DOI:** 10.1128/spectrum.00775-23

**Published:** 2023-10-05

**Authors:** Lisa Perruzza, Carlo Zagaglia, Laura Vitiello, Meysam Sarshar, Francesco Strati, Martina Pasqua, Fabio Grassi, Mauro Nicoletti, Anna Teresa Palamara, Cecilia Ambrosi, Daniela Scribano

**Affiliations:** 1 Institute for Research in Biomedicine, Faculty of Biomedical Sciences, Università della Svizzera Italiana, Bellinzona, Switzerland; 2 Humabs BioMed, a subsidiary of Vir Biotechnology, Bellinzona, Switzerland; 3 Department of Public Health and Infectious Diseases, Sapienza University of Rome, Rome, Italy; 4 Laboratory of Flow Cytometry, IRCCS San Raffaele Roma, Rome, Italy; 5 Research Laboratories, Bambino Gesù Children’s Hospital, IRCCS, Rome, Italy; 6 Mucosal Immunology Lab, Department of Biotechnology and Biosciences, University of Milano-Bicocca, Milan, Italy; 7 Institute Pasteur Italy, Department of Biology and Biotechnologies "Charles Darwin", Sapienza University of Rome, Rome, Italy; 8 Department of Public Health and Infectious Diseases, Sapienza University of Rome, Laboratory Affiliated to Institute Pasteur Italia-Cenci Bolognetti Foundation, Rome, Italy; 9 Department Infectious Diseases, Istituto Superiore di Sanità, Rome, Italy; 10 Department of Human Sciences and Quality of Life Promotion, San Raffaele University, Rome, Italy; 11 Laboratory of Microbiology of Chronic-Neurodegenerative Diseases, IRCCS San Raffaele Roma, Rome, Italy; Indian Institute of Science, Bangalore, Karnataka, India

**Keywords:** *Shigella flexneri*, apyrase, ATP, pyroptosis, inflammation, host cell survival

## Abstract

Intestinal epithelial cells represent the first line of defense from invading enteric pathogens. During the course of infection, pro-inflammatory programmed cell death is an effective way to eliminate invading microbes and to create a localized inflammatory environment. On the other hand, pathogens evolved countless strategies to overcome cell death and to keep the host alive ensuring their spread. It was previously shown that *Shigella flexneri* apyrase interacts with OmpA to contribute to a proper polar exposition of IcsA, which mediates actin-based motility. However, apyrase is also an ATP-diphosphohydrolase whose catalytic activity function has not been elucidated yet. Herein, we demonstrated that apyrase contributes to the manipulation of host cell fate by *S. flexneri* since it is released within the host cell cytoplasm during infection to degrade intracellular ATP. Thus, apyrase contributes to prevent caspase-1 activation, thereby downregulating the activation of pyroptosis in infected cells. Overall, apyrase is involved in the modulation of host cell survival and dampens the inflammatory response.

**IMPORTANCE:**

In this paper, we demonstrated that apyrase is released within the host cell cytoplasm during infection to target the intracellular ATP pool. By degrading intracellular ATP, apyrase contributes to prevent caspases activation, thereby inhibiting the activation of pyroptosis in infected cells. Our results show, for the first time, that apyrase is involved in the modulation of host cell survival, thereby aiding this pathogen to dampen the inflammatory response. This work adds a further piece to the puzzle of *Shigella* pathogenesis. Due to its increased spread worldwide, prevention and controlling strategies are urgently needed. Overall, this study highlighted apyrase as a suitable target for an anti-virulence therapy to tackle this pathogen.

## INTRODUCTION

Host–pathogen interaction is a highly dynamic process involving bacterial virulence factors and host defense mechanisms. When encountering the host defense system, numerous intracellular pathogens employ a variety of strategies evolved to escape, modulate, and hijack host innate immunity during infection (1, 2). Immune escape mechanisms are shared among “professional” as well as opportunistic bacterial pathogens, thereby allowing them to reside within the host (2, 3). *Shigella flexneri* is a facultative intracellular pathogen that in humans causes bacillary dysentery (4). Its pathogenicity relies on the expression of the type III secretion system (T3SS) that injects effector proteins inside host cells. These effectors induce bacterial invasion and are the main players in the modulation of the host innate immune response throughout the *Shigella* infection process (5, 6). Apyrase is encoded by the *phoN2* (*apy*) gene, which is located on a highly conserved region, the *ospB-phoN2* operon, within the pINV of *Shigella* species, and related enteroinvasive *Escherichia coli* (EIEC) strains (5). Apyrase (PhoN2) is a periplasmic ATP-diphosphohydrolase, which belongs to the family of the non-specific bacterial acid phosphatases (NSAPs) of class A. The expression of the *ospB-phoN2* operon is under the control of the regulatory network governing the expression of all *S. flexneri* virulence genes. In particular, the transcriptional factor VirF activates the expression of *icsA* and the secondary transcriptional activator, VirB; VirB, in turn, activates the expression of genes coding for the *Shigella* T3SS, for the early effectors, and for MxiE. MxiE, under T3SS active secretion, is responsible for the expression of late effectors and the upregulation of the *ospB-phoN2* operon (7 – 9). OspB is a T3SS effector, which modulates the host inflammatory response during the early stages of infection to promote bacterial dissemination (10). Vice versa, apyrase allows proper IcsA exposition at the old bacterial pole by interacting with the C-terminal domain of the outer membrane protein A (OmpA); proper exposition of IcsA ensures *Shigella* intra- and inter-cellular actin-based motility (11, 12). Indeed, the lack of apyrase resulted in a significant decrease in virulence due to impaired actin tail formation, as shown by reduced plaque size (13). However, we previously showed that this role is independent of its catalytic activity because catalytic-site mutants retained actin-based motility (11, 13). In fact, the function, if any, of the ATP-hydrolyzing activity during *S. flexneri* infection and pathology is still unknown.

Programmed cell death (PCD) is an energetically costly mechanism aimed at eliminating infected cells to restrict intracellular pathogen replication and spreading (14). This highly regulated process involves a number of ATP-dependent steps, such as caspase activation, enzymatic hydrolysis of macromolecules, chromatin condensation, and cell blebbing (15). Among them, pyroptosis is a pro-inflammatory form of PCD that very efficiently eradicates bacterial invaders. Upon cell death, a plethora of cytokines as well as alarmins, including ATP, are released to trigger a cascade of responses from the neighboring cells as well as to recruit immune cells (16 – 18). Hence, ATP is the key molecule bridging PCD and inflammation. Furthermore, it was shown that cells infected with different bacterial species, including *Shigella*, release ATP in the extracellular milieu as an early alert response to infection (18 – 21). Therefore, extracellular ATP (eATP) has gained recognition as an endogenous signaling molecule in several pathogenic processes, including bacterial infection (20, 21). Its extracellular concentration is negligible in healthy tissues because extracellular enzymes efficiently degrade it. Conversely, during bacterial infection, massive release of ATP modulates inflammation upon binding to purinergic receptors, ubiquitously expressed by human cells. eATP induces cell type-specific responses including NF-kB activation, expression of adhesion molecules and pro-inflammatory mediators, enhanced phagocytosis and phagocyte migration as well as activation of the inflammasome (20, 21). Canonical and non-canonical inflammasome activation, resulting in caspase-1 processing of pro-interleukin (IL)−1β and IL-18 as well as caspase-mediated cleavage of gasdermin D, lead to pyroptosis in both epithelial and immune cells (16).

*Shigella* exploits macrophage pyroptosis during the early stages of infection to gain entry into the intestinal submucosa (6, 22). Since activation of pyroptosis in epithelial cells at later stages of infection can lead to bacterial clearance (17, 22), *Shigella* evolved cell-type specific strategies to modulate this mechanism during host infection. Herein, we demonstrated that apyrase is involved in the manipulation of host cell fate since it is released within the host cell cytoplasm during infection to degrade intracellular ATP (iATP). Thus, apyrase contributes to prevent caspase-1 activation, thereby downregulating the activation of pyroptosis in infected cells. Taken together, our results show that apyrase is involved in the modulation of host cell survival and dampens host inflammatory response.

## MATERIALS AND METHODS

### Bacterial strains and cell line

*S. flexneri* wild-type M90T strain, the isogenic *apy* null mutant HND115 strain, and the complemented HND115(pHND10) strain carrying the gene *apy* fused with the HA tag (*apy*::HA) within the pHND10 plasmid (11) were plated on trypticase soy broth agar (TSA) plates (Gibco, Thermo Scientific, Milan, Italy) containing 0.01% Congo Red (CR) and grown on Luria-Bertani (LB) broth (Gibco, Thermo Scientific, Milan, Italy). The *mxiA* null mutant was cultured as described above and used as a negative control for T3SS secretion (13). When required, antibiotics were included at the following concentrations: ampicillin (Ap), 100 µg/mL; chloramphenicol (Cm), 30 µg/mL. L-arabinose was used at the final concentration of 0.016% to induce the expression of the fusion protein Apy-HA. The human cell line Caco-2 was cultured in Dulbecco’s modified Eagle’s medium (DMEM) supplemented with 10% fetal bovine serum (FBS, Gibco, Thermo Scientific, Milan, Italy) and grown in the presence of 5% CO_2_ at 37°C. *S. flexneri* invasion of semi-confluent monolayers was carried out at a multiplicity of infection (MOI) of 100, using the gentamicin protection assay, as previously described (10, 23). Intracellular bacterial counting, expressed as colony forming unit per mL (CFU/mL), was performed by cell lysis with 0.1% TritonX-100 for 5 min at RT. Cell lysates were serially diluted and plated onto LB agar plates.

### Quantification of intracellular ATP

Cell monolayers were infected with strains M90T, HND115, and HND115(pHND10); 3 hours post-infection (HPI), cells were treated with trypsin 0,25% (Gibco, Thermo Scientific, Milan, Italy), centrifuged 5 min at 1,500 × *g,* and lyzed with 100 µL of 0.1% TritonX-100 for 5 min at RT. Cell lysates were centrifuged at 5,000 × *g* to remove bacteria, and 10 µL of each supernatant was used to quantify intracellular ATP, using the Molecular Probes ATP determination Kit (Molecular Probes, Thermo Scientific, Milan, Italy) following the manufacturer’s instruction.

### Immuno-dot blot assay

Immuno-dot blot experiments were carried out as previously described (24). Briefly, exponentially grown and Congo Red-treated bacteria as well as bacteria recovered from infected cells were pelleted by centrifugation and re-suspended into equivalent volumes of PBS; 5 µL of each bacterial suspension was spotted onto a nitrocellulose membrane (Hybond-C, Millipore, Milan, Italy) and allowed to air dry. Membranes were then processed following standard Western blotting procedures using polyclonal sera: mouse anti-PhoN2, mouse anti-SurA, rabbit anti-IcsB, and rabbit anti-OmpA.

### Total protein extracts, gel electrophoresis, and Western blot analysis

M90T, *mxiA* mutant, and HND115(pHND10) strains were exponentially grown in 120 mL of LB medium at 37°C, and 60 mL of these cultures was induced with Congo Red to allow for T3SS secretion for 30 min at 37°C. Then, induced and non-induced bacteria were pelleted by centrifugation and re-suspended in equivalent volumes of 1X Leammli buffer, whereas bacterial supernatants were concentrated by precipitation in trichloroacetic acid (TCA). Protein samples were quantified by bicinchoninic acid assay (BCA) (Pierce BCA Protein Assay Kit, Thermo Fisher, Milan, Italy). Equal protein amounts were resolved by 12% SDS-PAGE and electrotransferred onto PVDF membranes (GE-Healthcare, Thermo Scientific, Milan, Italy). Membranes were probed with polyclonal anti-PhoN2 and anti-IcsB antibodies.

Cells were lyzed with 0,1% TritonX-100 for 5 min at RT. Cell lysates were centrifuged at 5,000 × *g* to remove bacteria, and after the addition of 1 mM PMSF, lysates were concentrated by using the 10 and 100 kDa cut off Vivaspin concentrators (Sartorius Italy S.r.l., Turin, Italy). Cell samples were re-suspended in 5X Laemmli buffer and diluted to 3 µg/µL; after denaturation for 10 min at 95°C, equal protein amounts were resolved by 12% SDS-PAGE and electrotransferred onto PVDF membranes. Membranes were probed with polyclonal anti-PhoN2, anti-SurA, anti-IpaB, and anti-OmpA sera and with anti-*E*. *coli* RNA Sigma 70 monoclonal antibody (MyBioSource, Aurogene Srl, Rome, Italy). For cell death marker detection, non-infected and infected cells, at 3 HPI, were lyzed in 1X Laemmli buffer and diluted to 3 µg/µL, whereas supernatants collected at 16 HPI were treated as described above. Whole cell extracts (WCEs) and supernatants (150 µg/lane) were assayed for caspase-1, caspase-3, gasdermin D, and IL-1β by Western blot using mouse monoclonal anti-caspase-1, goat polyclonal anti-caspase-3, and mouse monoclonal anti-gasdermin D antibodies from Santa Cruz Biotechnology (Santa Cruz Biotechnology, Milan Italy); rabbit monoclonal anti-IL-1β (ABclonal, Aurogene Srl, Rome, Italy); and rabbit polyclonal anti-GAPDH (Bethyl, SIAL Srl, Rome, Italy) antibodies. Appropriate secondary antibody anti-IgG conjugated to horseradish peroxidase were used (Bio-Rad, Milan, Italy). Blots were visualized by an enhanced chemiluminescence system (Pierce, Thermo Scientific, Milan, Italy). Relative band intensities were quantified by densitometric analysis using the ImageJ software as previously described (10, 25).

### Caspases activity, AnnexinV/PI and IL-1β staining, and FACS analysis

At 3 HPI, control and infected cells were treated with trypsin, collected and assayed for caspases activity using the Vybrant FAM poly Caspase Assay Kit (Molecular Probes, Thermo Scientific, Milan, Italy), following the manufacturer’s instructions. Annexin V/PI staining (eBioscience Annexin V Apoptosis Detection Kits, Invitrogen, Thermo Scientific, Milan, Italy) was performed following the manufacturer’s instructions. At 16 HPI, control and infected cells were treated with trypsin, collected and analyzed for IL-1β production, using BD Cytofix/Cytoperm Kit (BD Biosciences, Becton Dickinson Italia S.p.A., Milan, Italy) and the mouse anti-human IL-1β Alexa Fluor647 antibody (BD Pharmingen, Becton Dickinson Italia S.p.A., Milan, Italy), following the manufacturer’s instructions. Samples were washed twice in PBS, in Annexin V Buffer, or in BD Perm/Wash Buffer (BD Biosciences, Becton Dickinson Italia S.p.A., Milan, Italy) and acquired using the LSRFortessa X20 flow cytometer (BD Biosciences, Becton Dickinson Italia S.p.A., Milan, Italy); data were analyzed using FACS Diva software (v 8.02, BD Biosciences).

### Statistical analyses

Normal distribution was determined by the Shapiro–Wilk test. The statistical differences of normally distributed data were analyzed by one-way ANOVA, two-way ANOVA, and post-hoc Student’s *t*-test by using GraphPad Prism 7.00 software (San Diego, USA). Values of *P* < 0.05 were taken as being statistically significant.

## RESULTS

### Apyrase hydrolyzes extracellular ATP during bacterial growth

**Fig 1:**
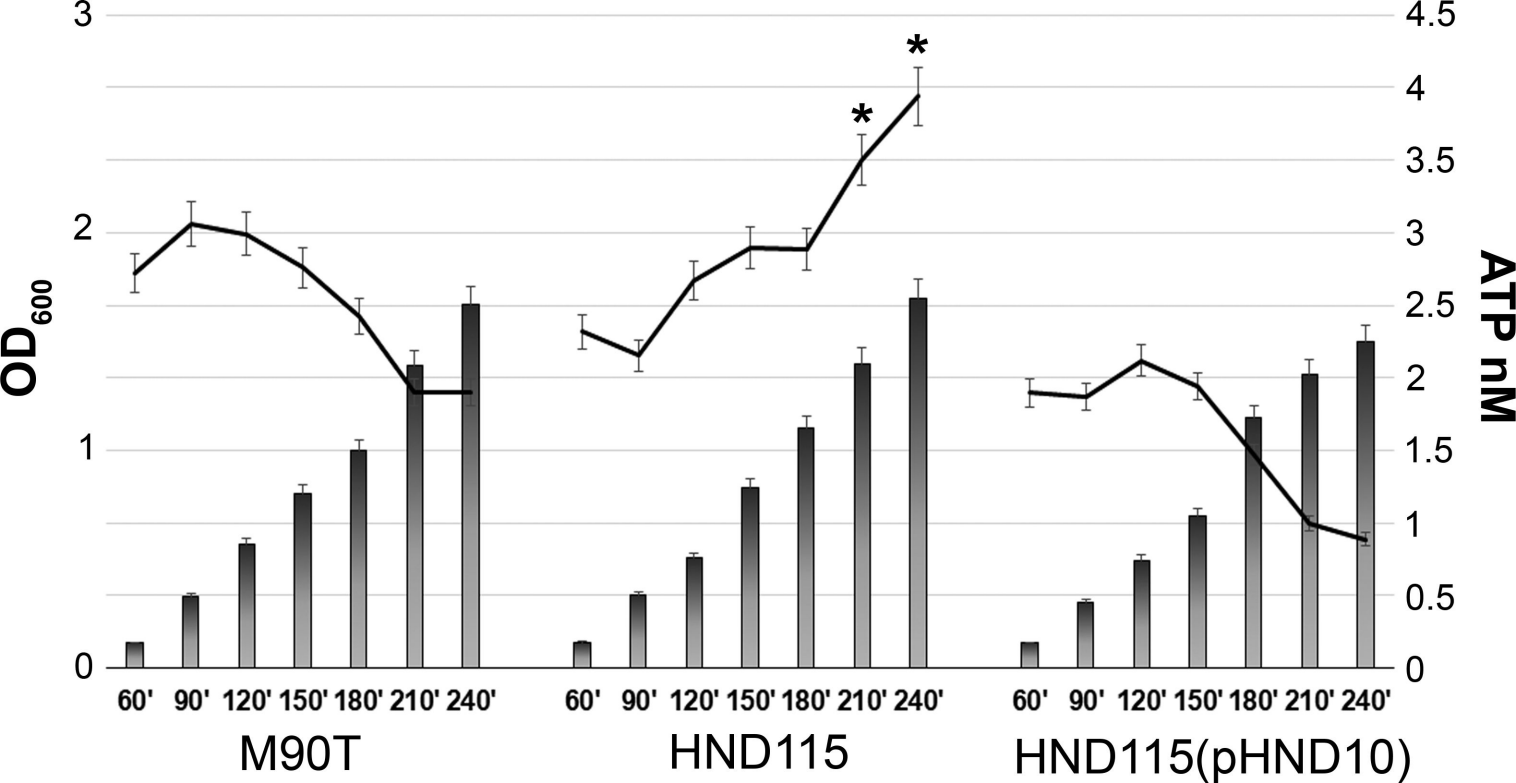
Apyrase hydrolyzes extracellular ATP. M90T, HND115, and HND115(pHND10) strains were cultivated in LB at 37°C for 4 hours; aliquots of bacterial cultures and relative supernatants were used to determine the OD_600_ (bars) and the extracellular ATP amount (line curves), respectively. Results represent means ± standard deviation (SD) of three independent experiments. Asterisks represent *P* values evaluated by two-way ANOVA, *P < 0.05. ATP values were statistically significantly higher in the supernatant from strain HND115 compared to those obtained from M90T and HND115(pHND10) strains at 210 and 240 min of bacterial growth.

To evaluate whether the periplasmic apyrase could degrade eATP during bacterial growth, the wild-type strain M90T, the isogenic apyrase null mutant HND115, and the complemented HND115(pHND10) strain expressing the fusion protein Apy-HA (11) were cultivated in LB at 37°C for 4hours (mid-exponential phase), and eATP was quantified every 30 min. Figure 1 shows that the levels of eATP in the supernatants from the HND115 strain were higher compared to those from the wild-type and complemented strain, suggesting that periplasmic apyrase is able to degrade eATP. Moreover, no difference in the growth rates of the three strains was observed, indicating that the hydrolysis of eATP does not manifestly impact bacterial metabolism. To test if apyrase could be released in the extracellular environment, M90T, *mixA* mutant, and HND115(pHND10) strains were cultivated in LB at 37°C in the absence and presence of Congo Red, a dye known to induce the opening of the T3SS (26). The *mixA* mutant was added as a T3SS negative control, being MxiA critical for the secretion of the invasion proteins (13). Aliquots collected from each condition were centrifuged, filtered, concentrated, and used to detect apyrase by Western blot. Any attempts to visualize apyrase in the supernatants failed, suggesting that apyrase is released by *S. flexneri* neither during its growth at 37°C nor under active secretion conditions (Fig. S1). Apyrase was slightly detectable only in the supernatant collected from the CR-induced complemented strain [HND115(pHND10)], probably due to protein expression not at physiological levels.

### Apyrase targets host-cell ATP

**Fig 2:**
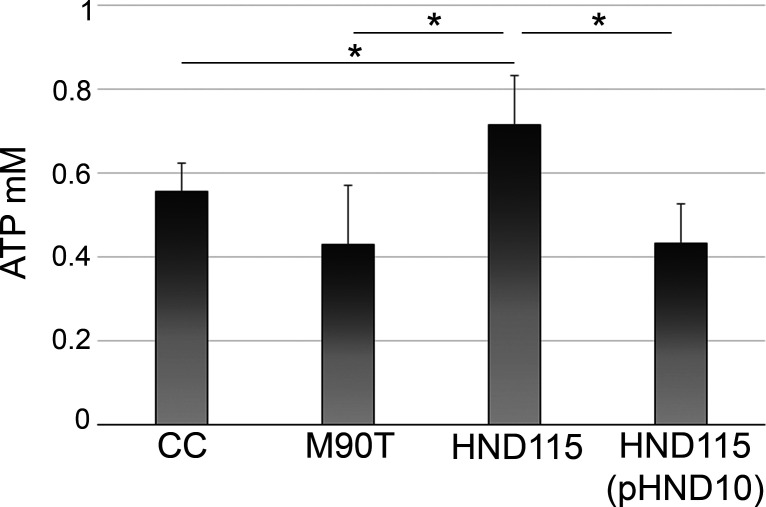
Apyrase reduces ATP content in infected cells. Cell monolayers were infected with strains M90T, HND115, and HND115(pHND10); at 3 hours post-infection, non-infected (CC) and infected cells were collected and lyzed with 100 µL of 0,1% TritonX-100 for 5 min at RT. Cell lysates were centrifuged at 5,000 × *g* to remove bacteria, and 10 µL of each supernatant was used to quantify intracellular ATP. Results represent means ± standard deviation of four independent experiments. Asterisks represent *P* values evaluated by one-way ANOVA Tukey’s multiple comparisons test, *P < 0.05.

We reasoned that apyrase could hydrolyze ATP within host cells during the bacterial infectious process. Hence, M90T, HND115, and HND115(pHND10) strains were used to infect Caco-2 cells for 3 hours. At this time point, both infected cells and related supernatants were lyzed and collected, respectively, for ATP quantification. Interestingly, a higher level of iATP was observed in cells infected with strain HND115 compared to cells infected with M90T and HND115(pHND10) strains and the non-infected control (Fig. 2). Vice versa, no significant difference was observed by comparing the levels of ATP in supernatants collected from infected and non-infected cells (data not shown). Moreover, the levels of iATP in cells infected with the wild-type strain and the complemented strain showed no significant difference compared to those observed in non-infected cells, suggesting that apyrase-proficient *S. flexneri* can control the iATP concentration in infected cells to maintain its levels at cellular physiological conditions (Fig. 2).

### Apyrase localizes on the bacterial surface and is released during cell infection

**Fig 3:**
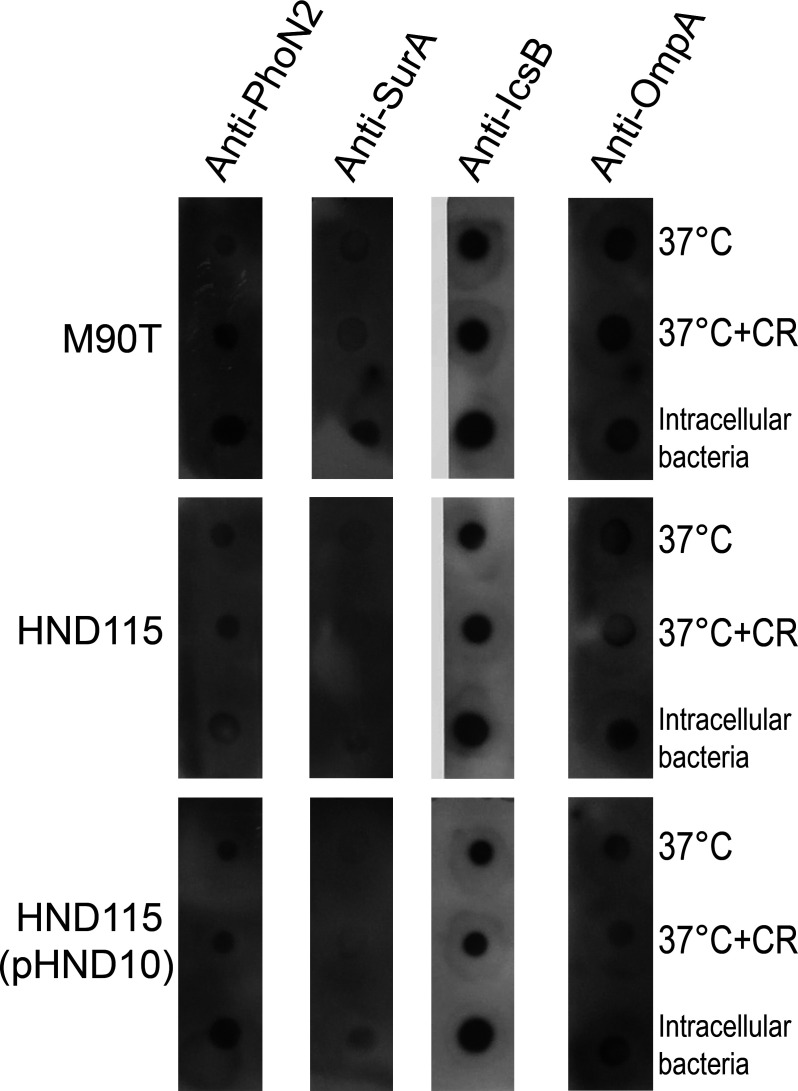
Apyrase is exposed on the bacterial surface. Dot blots of exponentially grown, Congo Red-treated as well as bacteria recovered from infected cells. For each condition, bacteria were pelleted by centrifugation and re-suspended in equivalent volumes of PBS. 5 µL of each bacterial suspension was spotted onto a nitrocellulose membrane and processed following standard Western blotting procedure, using polyclonal anti-PhoN2, anti-SurA, anti-IcsB, and anti-OmpA antibodies.

**Fig 4:**
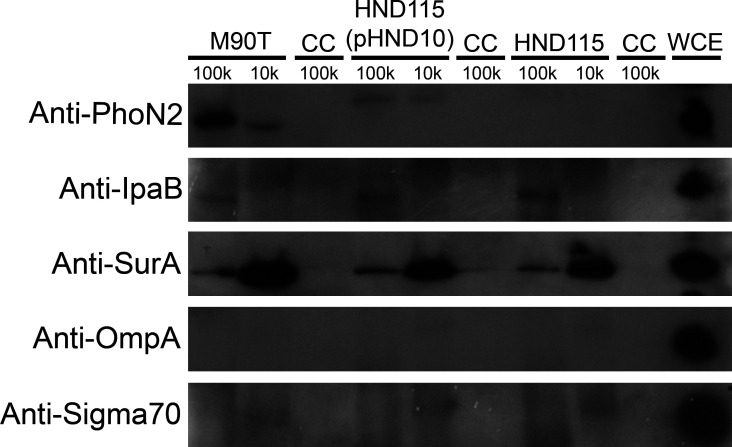
Apyrase localizes inside host cells. Cell monolayers were infected with strains M90T, HND115, and HND115(pHND10). Whole cell extract from strain M90T grown at 37°C was used as a positive control. At 4 hour post-infection, non-infected and infected cells were lyzed with 0,1% TritonX-100 for 5 min at RT, and lysates were concentrated using 100 kDa and 10 kDa cut-off concentrators. Proteins were resolved by 12% SDS-PAGE and electrotransferred onto PVDF membranes. Membranes were probed with polyclonal anti-PhoN2, anti-SurA, anti-IpaB, and anti-OmpA sera and with anti-*E*. *coli* RNA Sigma70 monoclonal antibody. The higher molecular weight of apyrase detected in strain HND115(pHND10) is due to the HA tag fused to the coding sequence of apyrase.

The observed hydrolase activity on iATP prompted us to analyze if apyrase was exposed on the outer membrane of *S. flexneri*. To test this hypothesis, an immuno-dot blot assay on intact bacteria was performed. Bacteria grown in LB at 37°C in the absence and presence of CR as well as those recovered from infected cells were re-suspended in PBS and spotted onto nitrocellulose membranes. Membranes were hybridized with anti-PhoN2, anti-SurA, anti-OmpA, and anti-IcsB polyclonal sera (Fig. 3). Notably, apyrase was detected on the bacterial external surface as OmpA and IcsB proteins, which were used as markers of surface and injected proteins, respectively. Vice versa, the chaperone SurA, included as a periplasmic marker, was barely, if at all detectable, indicating that spotted bacteria were still intact (Fig. 3). Then, we addressed whether apyrase could also be released into the host cell cytoplasm by intracellular bacteria during infection. To test this hypothesis, Caco-2 cells were infected with strains M90T, HND115, and HND115(pHND10) and lyzed at 4 HPI. Cell lysates were extensively centrifuged to remove intracellular bacteria, and total protein extracts were concentrated (100 and 10 kDa cut-off), subjected to SDS-PAGE, and hybridized with the same antibodies used in the immuno-dot blot assay; anti-sigma70 antibody was used as an additional internal control for the detection of intracellular bacterial proteins. Remarkably, apyrase was found in the cytoplasmic fraction of Caco-2 cells (Fig. 4), suggesting that it is released by bacteria during infection. In addition to apyrase, also SurA was detected in the protein extracts, indicating that intracellular bacteria can release their periplasmic content into the mammalian cytosol, during cell division and/or cell lysis (Fig. 4). It is important to underline that this chaperone is highly expressed during bacterial growth due to its activity on outer membrane protein assembly (27). As expected, the effector IpaB was detected in the cytoplasmic fraction of Caco-2 cells (5), confirming the delivery of *S. flexneri* secreted proteins within host cells (Fig. 4). OmpA was not detected in the protein extracts probably due to its precipitation with the insoluble fraction. Finally, Sigma70 was barely detected in the protein extracts, indicating negligible level of contamination by bacterial cytoplasmic proteins within the host cytosol. Altogether, these results indicate that apyrase localizes on the external surface of the bacterium, and during the infection it is released into the host cell, reducing the host cytoplasmic ATP pool.

### Apyrase downregulates caspase-1 activity and promotes cell survival

**Fig 5:**
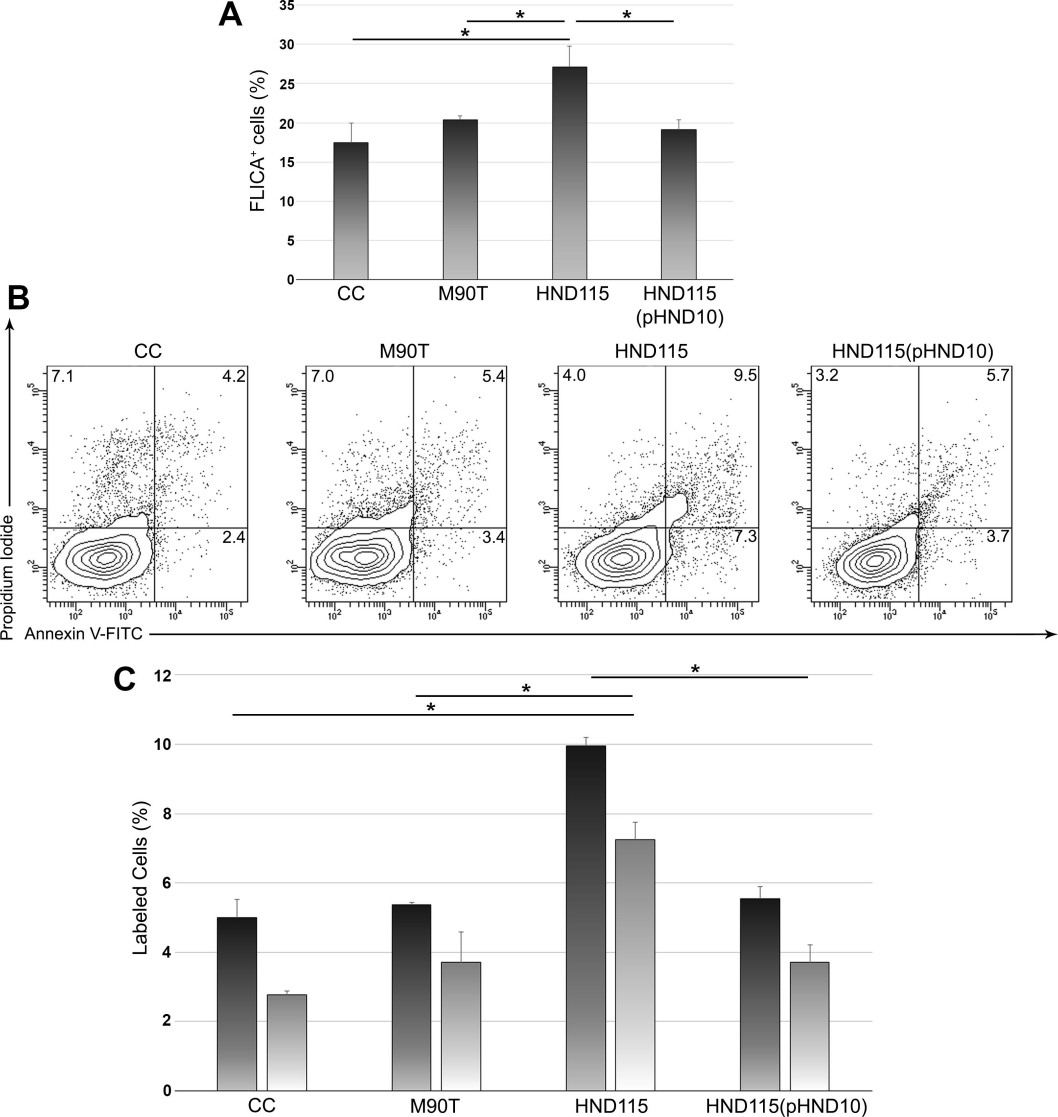
Apyrase downregulates caspases activity and delays cell death. Cell monolayers were infected with strains M90T, HND115, and HND115(pHND10). At 3 hours post-infection, non-infected and infected cells were collected and stained with Fluorochrome-Labeled Inhibitors of Caspases (FLICA) reagent or with AnnexinV/PI staining for 1 hour and 15 min, respectively. Cells were washed with PBS or Annexin V binding buffer and acquired with LSRFortessa X20 flow cytometer; data were analyzed using FACS Diva software. (**A**) Percentage of FLICA-positive cells. (**B**) Representative contour plots of Annexin V/PI stained infected and non-infected cells. Numbers in each quadrant represent the percentage of positive cells. (**C**) Bars show the quantification of Annexin V-positive cells (dark gray) and AnnexinV/PI double-positive cells (light gray) expressed as means ± SD of four independent experiments. Asterisks represent *P* values evaluated by one-way ANOVA Tukey’s multiple comparisons test, *P < 0.05.

**Fig 6:**
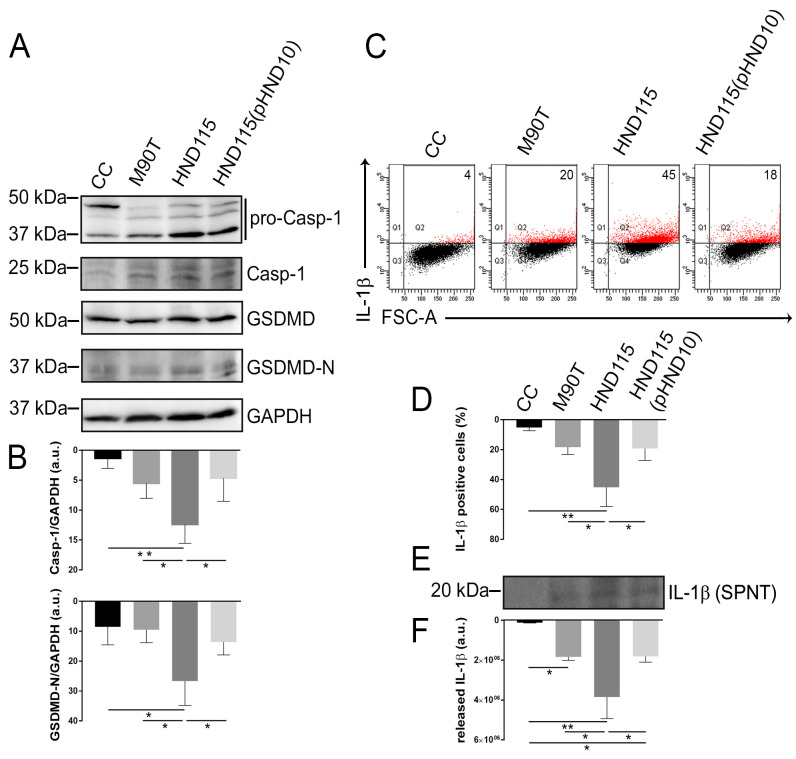
Apyrase prevents caspase-1 activation, leading to gasdermin D and pro-IL-1β processing arrest. (**A**) Cell monolayers were infected with strains M90T, HND115, and HND115(pHND10). At 3 hours post-infection, non-infected and infected cells were lyzed, and equal protein amounts were resolved by 12% glycine SDS-PAGE, electrotransferred onto PVDF membranes, and probed with monoclonal anti-caspase-1, gasdermin D (GSDMD), and polyclonal anti-GAPDH antibodies. Representative Western blot images from three independent experiments are shown. (**B**) Cleaved caspase 1 and processed gasdermin D-N (GSDMD-N) were normalized to the housekeeping GAPDH protein and analyzed by densitometry. In parallel, cell monolayers and supernatants (SPNT) from non-infected and infected cells were collected at 16 HPI. (**C**) Cells were stained with the anti-IL-1β antibody for 1 hour, washed with BD Perm/Wash buffer, and acquired with LSRFortessa X20 flow cytometer. Data were analyzed using FACS Diva software. Numbers in each quadrant represent the percentage of positive cells. (**D**) Bars show the quantification of IL-1β-positive cells expressed as means ± SD of six independent experiments. (**E**) Concentrated cell supernatants were analyzed by Western blot using an IL-1β antibody. (**F**) Quantification of IL-1β released into the supernatant was achieved by densitometry. Data are expressed as arbitrary units and are means ± SD. Asterisks represent *P* values evaluated by post hoc student’s *t*-test and one-way ANOVA Tukey’s multiple comparisons test, *P < 0.05, **P < 0.01.

The activation of caspases is a high energetic-cost process leading to PCD (17). This mechanism is commonly activated by infected cells, representing a way to block the progression of infection (14). To understand whether apyrase could contribute to protract bacterial infection by limiting caspases activation, we analyzed the total caspases activity at 3 HPI. Interestingly, we observed a significant increase of caspases activity in cells infected with strain HND115 compared to the wild-type, the complemented strain and the non-infected control cells (Fig. 5A), thus suggesting that apyrase negatively influenced caspase activation in infected cells. Furthermore, in our experimental conditions, we did not detect differences in cleaved caspase-3 levels in infected and non-infected cells, suggesting a lack of apyrase-mediated impact on caspase-3 cleavage (Fig. S2). Then, the contribution of caspases-mediated cell death in Caco-2 cells infected with M90T, HND115, and HND115(pHND10) strains was evaluated by Annexin V and PI staining analyzed by flow cytometry. Indeed, since PI enters death cells, it is used to identify necrotic cells, as defined by loss of plasma and nuclear membranes integrity; conversely, the Annexin V signal recognizes surface exposed phosphatidylserine, which is specific for the detection of early apoptotic cells, although it marks also pyroptotic cells. Several studies propose that double stained cells are indicative of the pyroptotic-mediated cell death (28 – 30). Results showed a significant increase in the number of Annexin V-positive as well as Annexin V/PI-positive cells in cells infected with strain HND115 (Fig. 5B and C) suggesting the activation of pyroptosis-dependent cell death. Hence, to evaluate if we could detect any reduction of intracellular bacteria in infected cells since dying cells release bacteria in the medium containing the antibiotic, we quantified the number of intracellular bacteria at the same time point of Annexin V/PI staining (3 HPI). Interestingly, we observed about 1 log reduction in the CFU/mL of strain HND115 compared to the wild-type and the complemented strain (Fig. S3). No differences in invasion rates were observed among strains (data not shown). Furthermore, to corroborate the induction of pyroptosis, we analyzed the extent of caspase-1 and gasdermin D activation in infected cells at 3 HPI by Western blot. Results showed statistically significantly higher levels of cleaved caspase-1 and processed form of gasdermin D in cells infected with strain HND115 compared to those infected with the wild-type, the complemented strain, and the non-infected controls (Fig. 6A and B). Intracellular and released IL-1b levels were also quantified at 16 HPI by flow cytometry and Western blot, respectively. A higher percentage of IL-1β-positive cells were found in cells infected with strain HND115 compared to the wild-type, the complemented strain, and the non-infected control cells (Fig. 6C and D). Accordingly, statistically significantly higher levels of IL-1β were detected in supernatants collected from cells infected with the mutant strain with respect to those obtained from cells infected with the wild-type and the complemented strain (Fig. 6E and F). Altogether, these results indicate that apyrase, by reducing the iATP content, limits caspase-1 activity, contributing to halting the pyroptotic mediator gasdermin D as well as pro-IL-1β processing. This activity results in the reduction of host cell death rate, thereby granting the intracellular blooming of bacteria.

## DISCUSSION

Intestinal epithelial cells represent the first line of defense from invading enteric pathogens. During the course of infection, pro-inflammatory PCD is an effective way to eliminate invading microbes and to create a localized inflammatory environment. On the other hand, pathogens evolved countless strategies to overcome cell death and keep the host alive ensuring their spread. Herein, we demonstrated that *S. flexneri* releases apyrase within the host cell cytoplasm during infection; due to its ATP-hydrolyzing activity, apyrase degrades iATP, thereby reducing the cell death rate of infected cells via preventing caspase-1 activation. Apyrase belongs to class A NSAPs, whose members are widely expressed among bacteria and are mainly associated with inorganic phosphate scavenging (31, 32). Class A enzymes displayed high variability in both activity and substrate recognition (31 – 33). It was reported that these small enzymes can be released in the extracellular environment, either in soluble form or retained as membrane-bound proteins where they can dephosphorylate a broad array of structurally unrelated phosphoester substrates (19, 31). Accordingly, apyrase could be visualized on the bacterial cell surface both during Congo Red induction of T3SS and from infected cells but not free in bacterial supernatants, indicating that it belongs to those NSAPs of class A that are exposed extracellularly (Fig. 3). Noteworthy, translocation on the surface of bacteria was linked to a significant hydrolysis of eATP in bacterial cultures as well as iATP in infected cells (Fig. 1 and 2). To the best of our knowledge, this is the first report describing that apyrase from *S. flexneri* degrades the host iATP pool within the host cytoplasm. Other bacterial acid phosphatases were shown to be involved in the virulence of several pathogens, although their exact role is still debated (33). These include SapM from *Mycobacterium tuberculosis*, involved in the subversion of the phagosomal maturation pathway in macrophages (34), several acid phosphatases from *Francisella tularensis* and *Francisella novicida* involved in macrophage survival (35), and an unidentified acid phosphatase of *Coxiella burnetii* that enhances bacterial survival by inhibiting the release of reactive oxygen intermediates by polymorphonuclear cells (36). Some of these acid phosphatases were found to be localized in the cell cytoplasm where they can target multiple substrates, but there is no evidence that these enzymes target iATP (37, 38). In general, enzymes with nucleosidase activity are shown to play several roles in bacterial virulence as well as in host colonization. Well-known examples of these enzymes include nucleoside diphosphate kinases secreted by extracellular as well as intracellular pathogens such as *Pseudomonas aeruginosa, Porphyromonas gingivalis,* and *M. tuberculosis* (39, 40). Interestingly, these enzymes hydrolyze ATP to ADP to regulate cell apoptosis/necrosis, phagocytosis, and the inflammatory response (39, 40). Hence, due to ATP’s master role in modulating critical cellular responses, bacteria evolved countless strategies to control intracellular and extracellular ATP content. In this context, it was shown that *Shigella* induces the opening of the connexin 26 and 43 hemichannels allowing release of ATP into the medium during the early stages of infection. Released ATP induces membrane ruffling of non-infected adjacent cells which, in turn, increases bacterial capture by filopodia, thereby enhancing bacterial internalization and spreading (18, 41). This strategy was one of the first demonstrations of how *Shigella* manipulates ATP-dependent signaling pathways (18, 41). However, it is well known that ATP represents also a danger-associated signaling molecule. In particular, eATP triggers inflammatory reactions by the activation of pyroptosis in infected cells, immune cell proliferation as well as massive pro-inflammatory cytokine release via the purinergic P2X7 receptor (20, 21). Therefore, exploiting ATP’s central role, *Shigella* is able to control its extracellular levels through the expression and release of the T3SS effector IpgD. By converting phosphatidylinositol 4,5-bisphosphate (PtdIns 4,5-P2) to PtdIns 5 P, IpgD increases PtdIns 5 P levels which, in turn, prevents hemichannel opening and ATP release (42). This event occurs early after infection, as demonstrated by high levels of eATP in cells infected with the *ipgD* null mutant, starting from 50 min post-infection onward. The final outcome was evidenced in the *in vivo* ileal loop model, where infection with the *ipgD* mutant induced a massive increase of pro-inflammatory cytokines and antimicrobial peptides, leading to severe destruction of the mucosa when compared to the infection with the wild-type strain (42). We observed significant differences in the amounts of iATP in cells infected with the apyrase mutant strain HND115 at 3 HPI, suggesting that apyrase might degrade iATP accumulating after IpgD-induced hemichannel closure. In line with this hypothesis, it was shown that co-injection of the *ipgD* mutant strain with an apyrase enzyme in the ileal loop model decreased the mucosal damage observed with the *ipgD* mutant alone (42). However, future experiments by using the double *ipgD-apy* null mutant will clarify if these two proteins are involved in the same pathway regulating the intracellular ATP amounts. Overall, fine-tuning of iATP levels represents the mechanism adopted by *Shigella* to maximize its residing within host cells. The intracellular lifestyle ensures protection from host immune response, limited competition with resident bacteria, and a highly rich nutrient environment. Interestingly, we did not observe any significant difference in iATP levels in cells infected with the wild-type strain and non-infected cells, suggesting that apyrase activity helps *S. flexneri* to keep physiological iATP levels during infection. Accordingly, it was reported that *Shigella* takes advantage of host metabolic sources while preserving the energy status of the host during infection to maintain its replication niche (43).

As stated before, there are several evidence highlighting that cells infected by intracellular pathogens as well as exposed to bacteria or bacterial toxins release ATP in the extracellular milieu, as shown by comparing the levels of eATP in infected vs non-infected cells (44). However, few data are available both on iATP quantification during infection and on the increase of iATP before PCD, absolutely required for its activation (15, 45). We observed higher levels of iATP and active caspases in cells infected with the HND115 strain at 3 HPI, compared to cells infected with the wild-type and the complemented strain. Accordingly, only one study addressed iATP levels during bacterial infection by a high-resolution analysis of ATP dynamic; results revealed that the increase of cytosolic ATP is associated with PCD in epidermal plant cells (45). Infection with the HND115 strain resulted in an increased number of AnnexinV/PI-positive cells compared to cells infected with the wild-type and the complemented strain (Fig. 5); furthermore, the lack of apyrase indirectly induced caspase-1 activation which, in turn, leads to gasdermin D cleavage and pro-IL-1β processing, thereby triggering pyroptotic cell death (Fig. 6). Pyroptosis was shown to be critical in controlling *Shigella* infection both *in vitro* and *in vivo* (6, 22, 46 – 48). Interestingly, Mitchell et al. demonstrated that activation of NAIP–NLRC4 inflammasome leads to pyroptosis of intestinal epithelial cells; this mechanism controls and coordinates the elimination of *Shigella*-infected epithelial cells, thereby restricting its spreading (48). Likewise, *Salmonella* infected epithelial cells are expelled from the intestinal epithelial barrier in an NLRC4-dependent manner (49). The principal bacterial factors that stimulate inflammasome assembly are the T3SS and the LPS. In particular, the needle proteins of the T3SS apparatus are bound by NAIP family members, while intracellular LPS is directly recognized and bound by human caspase-4 and caspase-5 (46, 47). Consequently, pyroptotic cell death, triggered by inflammasome activation, represents an effective host defense mechanism against bacterial infections connected with the immune system. In this context, it was recently demonstrated that *Shigella* copes with pyroptosis by secreting the T3SS OspC3 effector that catalyzes a novel type of posttranslational modification, the “ADP-riboxanation,” to modify specific arginine residues in human caspase-4 (Arg314) and murine caspase-11 (Arg310), thereby inhibiting pyroptosis (50). Furthermore, the ubiquitin ligase activity of the effector IpaH7.8 targets human gasdermin D, leading to its degradation, revealing another mechanism by which *Shigella* resists cell-intrinsic immunity to maintain its intracellular replicative niche (51). Here, we propose that apyrase should be considered as an additional regulator of host cell fate through its iATP degrading activity. Interestingly, apyrase was detectable as membrane-bound on intracellular bacteria at 3 HPI; conversely, it could be recovered and detected from the soluble fraction of infected cells at 4 HPI, leading to the hypothesis that it is released into the host cell cytoplasm at later time points. Since apyrase is not secreted by the T3SS, it is possible that a still unidentified mechanism releases it during the infectious process. Despite the possibility, it does not look plausible that released apyrase comes from bacterial lysis because we did not observe any decrease in intracellular bacterial counts at 4 HPI (data not shown). Therefore, we hypothesize that apyrase represents an additional *S. flexneri* moonlighting protein (52). Immediately after its translation, apyrase localizes in the periplasm beneath IcsA and promotes actin-based motility, then it translocates on the bacterial surface where it hydrolyzes eATP and then, during infection, it is released to target iATP. This long-lasting activity is supported by the high stability of *phoN2* mRNA, leading to its continuous expression during bacterial growth as well as during T3SS secretion (7, 8). Furthermore, it is well established that *Shigella* temporally controls the release of T3SS effectors. The early secretion lasts for 30–60 min upon entry; then, bacteria that escape from the entry vacuole become motile and reactivate their secretion system between 60 min and 120 min post-entry (53). Hence, during infection, it can be hypothesized that apyrase first mediates actin-based motility and, later on, modulates iATP content, thereby contributing to bacterial spread and host cell persistence in the intestinal epithelia.

**Fig 7:**
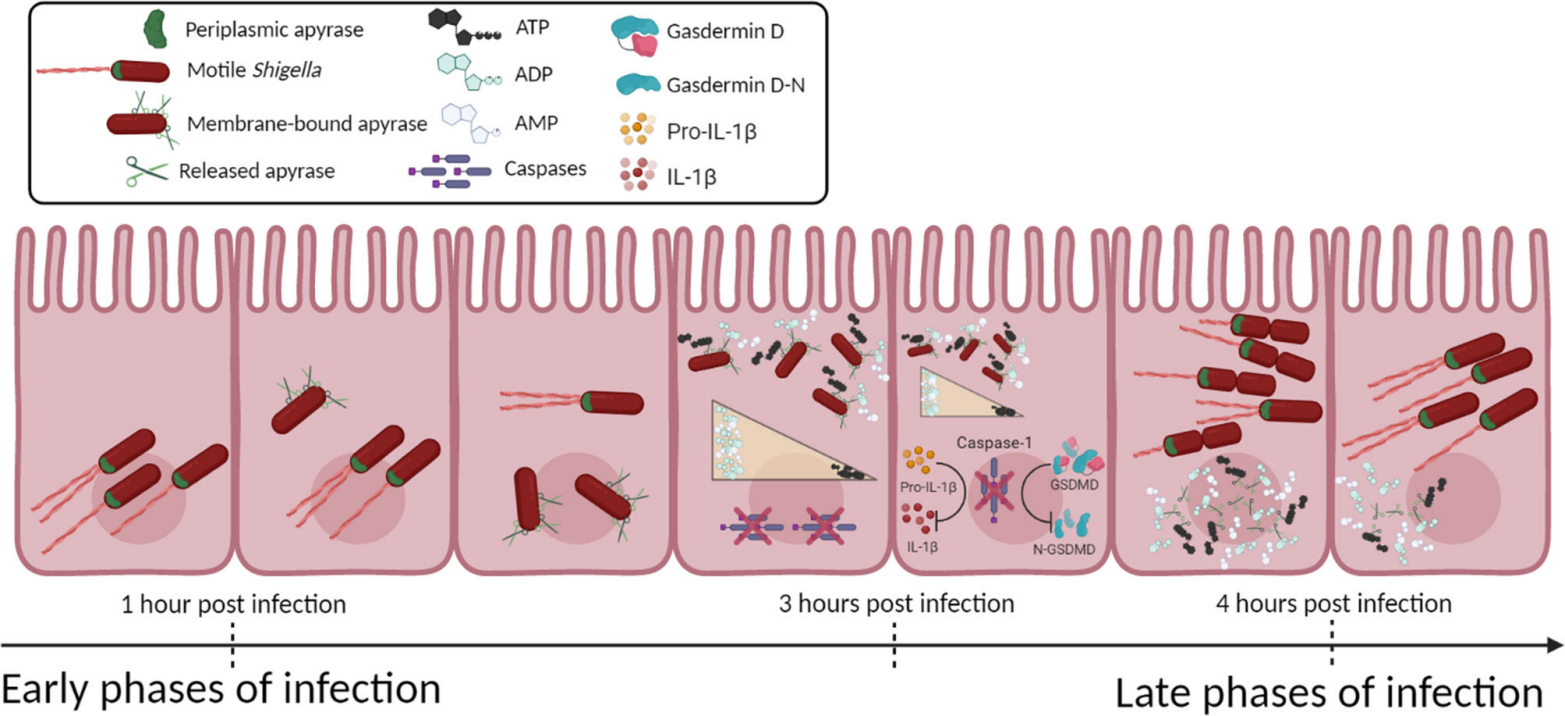
Working model of apyrase function during *S. flexneri* infection of intestinal epithelial cells. During the early phases of infection, apyrase mediates actin-based motility, by localizing at the old bacterial pole. Then, it translocates to the bacterial surface and, later on during infection, it is released inside the host cell cytoplasm degrading iATP. By reducing the iATP pool, apyrase helps *Shigella* to prevent caspase-1 activation, leading to the inhibition of ATP-mediated pyroptosis of infected cells, thereby promoting bacterial persistence and spread. Individual components are not to scale. Figure created with BioRender.com

In conclusion, our results indicate for the first time that the virulence factor apyrase controls the activity of the cellular central mediator ATP, by modulating iATP levels. Therefore, apyrase helps *Shigella* to downregulate caspase-1 activation contributing to halt gasdermin D and pro-IL-1β processing, leading to the inhibition of ATP-mediated pyroptosis in infected intestinal cells (Fig. 7). This virulence mechanism promotes *Shigella* residing within epithelial cells and hinders host inflammatory reaction, thereby allowing bacterial persistence and spread.

## SUPPLEMENTAL MATERIAL

Figure S1 spectrum.00775-23-s0001.tif Fig. S1 Apyrase is released neither during bacterial growth nor under active T3SS secretion. Exponentially-grown (37°C) and Congo Red (CR)-treated bacteria were pelleted by centrifugation and re-suspended in equivalent volumes of 1X Laemmli buffer, whereas bacterial supernatants were concentrated by TCA precipitation and quantified. Equal protein amounts were resolved by 12% SDS-PAGE and electrotransferred onto PVDF membranes. Membranes were probed with polyclonal anti-PhoN2 and anti-IcsB antibodies. The higher molecular weight of apyrase detected in strain HND115(pHND10) is due to the HA tag fused to the coding sequence of apyrase.

Figure S2 spectrum.00775-23-s0002.tif Fig. S2 Apyrase does not impact on caspase-3 cleavage. Cell monolayers were infected with strains M90T, HND115, and HND115(pHND10); at 3 hours post-infection cells were collected and equal amounts of proteins were subjected to Western blot assays using anti-caspase-3 and anti-GAPDH antibodies. Equal protein loading was confirmed by GAPDH detection.

Figure S3 spectrum.00775-23-s0003.tif Fig. S3 Apyrase contributes to preserve cell replicative niche. Cell monolayers were infected with strains M90T, HND115, and HND115(pHND10); at 3 hours post-infection (HPI) cells were lyzed with 0.1% Triton X-100 for 5 min at RT; lysates were serially diluted and plated onto LB agar plates for intracellular bacteria counting (CFU/ml). Graphs depict the means {plus minus} SD of six independent experiments; asterisks represent *P* values evaluated by post hoc Student's t-test, * *P* < 0.05.

Supplemental material legends spectrum.00775-23-s0004.docx Legends of Figs S1, S2 and S3.

ASM does not own the copyrights to Supplemental Material that may be linked to, or accessed through, an article. The authors have granted ASM a non-exclusive, world-wide license to publish the Supplemental Material files. Please contact the corresponding author directly for reuse.
