# Supplementary figures and images for: The Shigella flexneri virulence factor apyrase is released inside eukaryotic cells to hijack host cell fate
Source: Microbiol Spectr. 2023 Oct 5;11(6):e00775-23. doi: 10.1128/spectrum.00775-23 (PMC10714728; doi:10.1128/spectrum.00775-23)

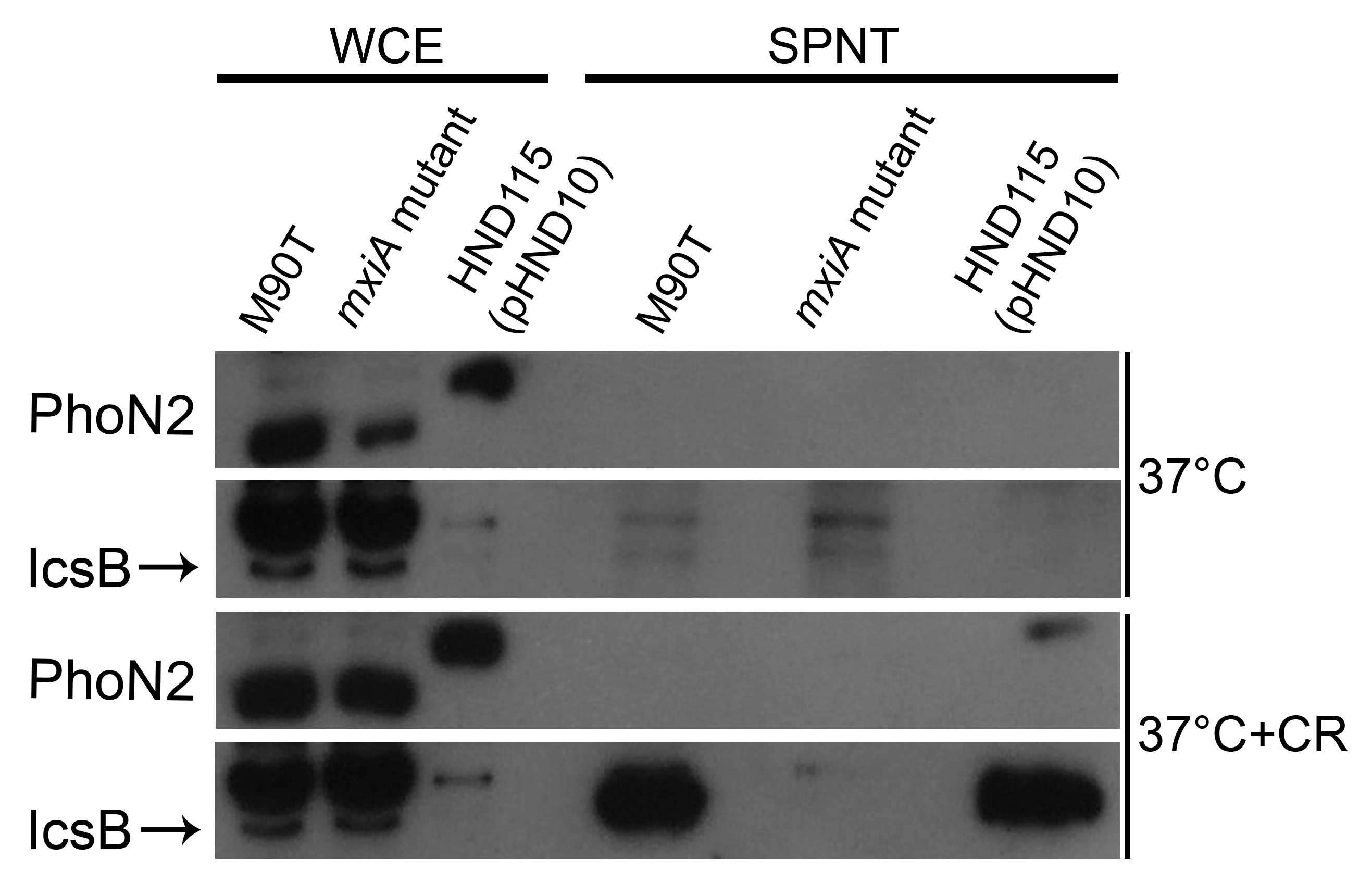

Supplement: Figure S1 — Fig. S1 Apyrase is released neither during bacterial growth nor under active T3SS secretion. Exponentially-grown (37°C) and Congo Red (CR)-treated bacteria were pelleted by centrifugation and re-suspended in equivalent volumes of 1X Laemmli buffer, whereas bacterial supernatants were concentrated by TCA precipitation and quantified. Equal protein amounts were resolved by 12% SDS-PAGE and electrotransferred onto PVDF membranes. Membranes were probed with polyclonal anti-PhoN2 and anti-IcsB antibodies. The higher molecular weight of apyrase detected in strain HND115(pHND10) is due to the HA tag fused to the coding sequence of apyrase. [file spectrum.00775-23-s0001.tif]

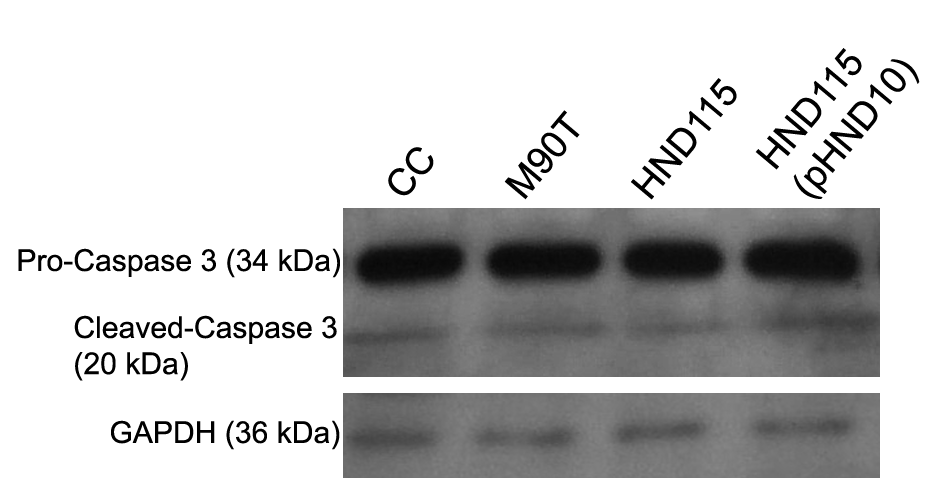

Supplement: Figure S2 — Fig. S2 Apyrase does not impact on caspase-3 cleavage. Cell monolayers were infected with strains M90T, HND115, and HND115(pHND10); at 3 hours post-infection cells were collected and equal amounts of proteins were subjected to Western blot assays using anti-caspase-3 and anti-GAPDH antibodies. Equal protein loading was confirmed by GAPDH detection. [file spectrum.00775-23-s0002.tif]

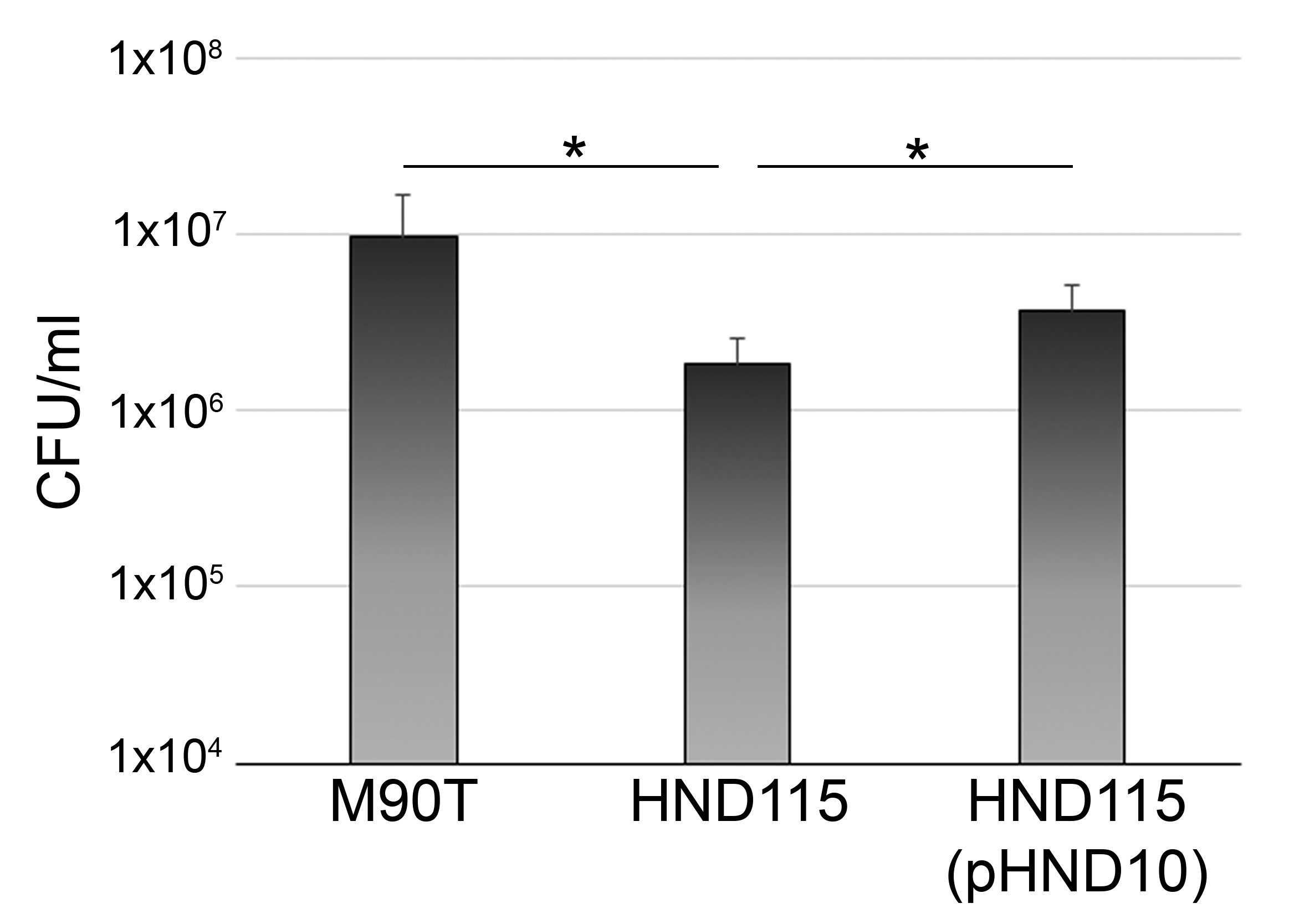

Supplement: Figure S3 — Fig. S3 Apyrase contributes to preserve cell replicative niche. Cell monolayers were infected with strains M90T, HND115, and HND115(pHND10); at 3 hours post-infection (HPI) cells were lyzed with 0.1% Triton X-100 for 5 min at RT; lysates were serially diluted and plated onto LB agar plates for intracellular bacteria counting (CFU/ml). Graphs depict the means {plus minus} SD of six independent experiments; asterisks represent P values evaluated by post hoc Student's t-test, * P < 0.05. [file spectrum.00775-23-s0003.tif]
