# Supplementary material for: The Shigella flexneri virulence factor apyrase is released inside eukaryotic cells to hijack host cell fate
Source: Microbiol Spectr. 2023 Oct 5;11(6):e00775-23. doi: 10.1128/spectrum.00775-23 (PMC10714728; doi:10.1128/spectrum.00775-23)
Supplement: Supplemental material legends — Legends of Figs S1, S2 and S3. [file spectrum.00775-23-s0004.docx]

**Fig. S1** **Apyrase is released neither during bacterial growth nor under active T3SS secretion.** Exponentially-grown (37°C) and Congo Red (CR)-treated bacteria were pelleted by centrifugation and re-suspended in equivalent volumes of 1X Laemmli buffer, whereas bacterial supernatants were concentrated by TCA precipitation and quantified. Equal protein amounts were resolved by 12% SDS-PAGE and electrotransferred onto PVDF membranes. Membranes were probed with polyclonal anti-PhoN2 and anti-IcsB antibodies. The higher molecular weight of apyrase detected in strain HND115(pHND10) is due to the HA tag fused to the coding sequence of apyrase.

**Fig. S2** **Apyrase does not impact on caspase-3 cleavage.** Cell monolayers were infected with strains M90T, HND115, and HND115(pHND10); at 3 hours post-infection (HPI) cells were collected and equal amounts of proteins were subjected to Western blot assays using anti-caspase-1 and anti-GAPDH antibodies. Equal protein loading was confirmed by GAPDH detection.

**Fig. S3 Apyrase contributes to preserve cell replicative niche.** Cell monolayers were infected with strains M90T, HND115, and HND115(pHND10); at 3 hours post-infection (HPI) cells were lyzed with 0,1% Triton X-100 for 5 min at RT; lysates were serially diluted and plated onto LB agar plates for intracellular bacteria counting (CFU/ml). Graphs depict the means ± SD of six independent experiments; asterisks represent *P* values evaluated by post hoc Student’s t-test, * *P* < 0.05.
